# Tetra­kis(μ-2-anilinobenzoato)bis­[methano­lcopper(II)](*Cu*—*Cu*)

**DOI:** 10.1107/S1600536808038063

**Published:** 2008-11-22

**Authors:** Chun-Wei Xin, Fu-Chen Liu

**Affiliations:** a School of Chemistry and Chemical Engineering, Tianjin University of Technology, Tianjin 300191, People’s Republic of China

## Abstract

The title compound, [Cu_2_(C_13_H_10_NO_2_)_4_(CH_4_O)_2_], has been prepared by the reaction of 2-anilinobenzoic acid, H*L*, with copper(II) nitrate in methanol. This dinuclear complex is arranged around an inversion center. Each Cu atom displays a distorted trigonal–pyramidal coordination with four O atoms from the four ligands *L* and one axial O atom of the methanol solvent mol­ecule. Each carboxyl­ate group of the ligands *L* links two Cu atoms, building a dinuclear complex with a Cu—Cu distance of 2.5774 (10) Å. There are intra­molecular N—H⋯O hydrogen bonds, and the H atom of the methanol mol­ecule is involved in weak bifurcated hydrogen-bonding inter­actions with two carboxyl­ate O atoms of related mol­ecules, forming a chain developing parallel to the *a* axis.

## Related literature

For general background, see: Melnik *et al.* (1998 ▶); Facchin *et al.* (1998 ▶); Martin & Greenwood (1997 ▶); Moulton *et al.* (2003 ▶). For a related structure, see: Churchill *et al.* (1985 ▶).
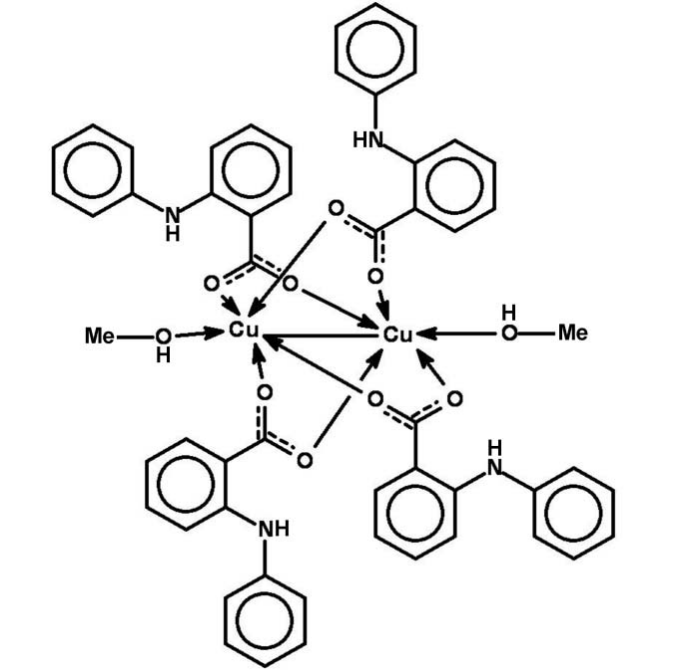


## Experimental

### 

#### Crystal data

[Cu_2_(C_13_H_10_NO_2_)_4_(CH_4_O)_2_]
*M* *_r_* = 1040.06
Monoclinic, 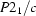

*a* = 7.2467 (14) Å
*b* = 14.171 (3) Å
*c* = 23.813 (5) Å
β = 97.11 (3)°
*V* = 2426.6 (9) Å^3^
*Z* = 2
Mo *K*α radiation
μ = 0.94 mm^−1^
*T* = 293 (2) K
0.22 × 0.20 × 0.15 mm

#### Data collection

Bruker SMART CCD diffractometer
Absorption correction: none
23688 measured reflections
5568 independent reflections
3886 reflections with *I* > 2σ(*I*)
*R*_int_ = 0.073

#### Refinement

*R*[*F* ^2^ > 2σ(*F* ^2^)] = 0.059
*wR*(*F* ^2^) = 0.115
*S* = 1.05
5568 reflections
316 parameters
H-atom parameters constrained
Δρ_max_ = 0.41 e Å^−3^
Δρ_min_ = −0.35 e Å^−3^

### 

Data collection: *SMART* (Bruker, 1998 ▶); cell refinement: *SAINT-Plus* (Bruker, 1998 ▶); data reduction: *SAINT-Plus*; program(s) used to solve structure: *SHELXS97* (Sheldrick, 2008 ▶); program(s) used to refine structure: *SHELXL97* (Sheldrick, 2008 ▶); molecular graphics: *ORTEPIII* (Burnett & Johnson, 1996 ▶) and *ORTEP-3 for Windows* (Farrugia, 1997 ▶); software used to prepare material for publication: *SHELXL97*.

## Supplementary Material

Crystal structure: contains datablocks global, I. DOI: 10.1107/S1600536808038063/dn2398sup1.cif

Structure factors: contains datablocks I. DOI: 10.1107/S1600536808038063/dn2398Isup2.hkl

Additional supplementary materials:  crystallographic information; 3D view; checkCIF report

## Figures and Tables

**Table 1:** Hydrogen-bond geometry (Å, °)

*D*—H⋯*A*	*D*—H	H⋯*A*	*D*⋯*A*	*D*—H⋯*A*
N1—H1⋯O2	0.83	2.04	2.690 (4)	135
N2—H2⋯O3	0.83	2.05	2.688 (4)	133
O5—H5*A*⋯O1^i^	0.84	2.54	3.306 (4)	152
O5—H5*A*⋯O4^i^	0.84	2.55	3.260 (4)	143

Symmetry code: (i) 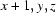.

**Fig. 1.**
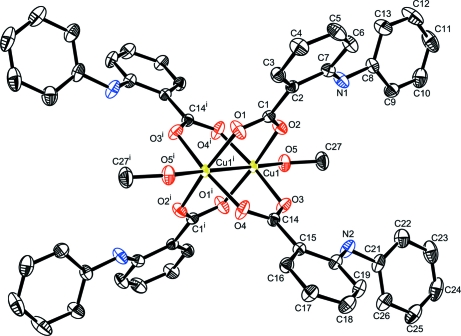
The molecular structure of the dinuclear complex with the atom-labelling scheme. Ellipsoids are drawn at the 30% probability level. H atoms have been omitted for clarity. [Symmetry code: (i) -x+2, -y, -z+2]

## supplementary crystallographic information

### Comment

There is an increasing interest in the design of metal complexes based on polydentate ligands(Martin & Greenwood, 1997). 2-anilinobenzoato and its derivatives with multifunctional sites can bridge metal ions in different mode allowing a large variety of structures (Melnik *et al.*, 1998). In the copper carboxylate based complexes dinuclear tetracarboxylate paddlewheel clusters have been frequently observed (Moulton *et al.*, 2003 and references therein). Several dimer complexes having similar structure to the title complex were reported (Facchin *et al.*, 1998 and references therein).

The dinuclear copper complex is built up around inversion center. Each copper atom displays a trigonal-bipyramidal coordination with four oxygen atoms from the four ligands *L* and one axial methanol solvent. Each carboxylate groups of the ligands *L* link two Cu atoms building a dinuclear complex (Fig. 1) with a Cu-Cu distance of 2.5774 (10) Å , typical of tetracarboxylate paddlewheel Cu dinuclear complex(Churchill *et al.*, 1985).

There are intramolecular N-H···O hydrogen bond whereas the H atom of the methanol is in weak bifurcated interactions with two carboxyalte O atoms of related molecule forming a chain developping parallel to the a axis (Table 1).

### Experimental

The title compound was prepared by adding 10 ml of methanol solution of copper nitrate (1 mmol) to 10 ml of methanol solution of *L*(0.5 mmol) neutralized by sodium aeide(1 mmol). The mixture was stirred for about 2 h and filtered.The filtrate was slowly evaporated at room temperture to yield cubic black crystals of (I) suitable for X-ray analysis. Yield 30% based on copper(II).

### Refinement

The H atoms attached to C atoms were included in calculated positions and treated as riding on their parent atoms with C—H = 0.93 Å(aromatic) or 0.96Å (methyl) with *U*_iso_(H) = 1.2*U*_eq_(C_aromatic_) or *U*_iso_(H) = 1.5*U*_eq_(C_methyl_) . The H atoms attached to N and O atoms were initially refined using N-H or O-H restraints (0.83 (2)Å), then they were treated as riding on their parent atoms in the last cycles of refinement with *U*_iso_(H) = 1.2*U*_eq_(N) or *U*_iso_(H) = 1.5*U*_eq_(O).

### Crystal data

**Table:**

[Cu_2_(C_13_H_10_NO_2_)_4_(CH_4_O)_2_]	*F*_000_ = 1076
*M**_r_* = 1040.06	*D*_x_ = 1.423 Mg m^−^^3^
Monoclinic, *P*2_1_/*c*	Mo *K*α radiation λ = 0.71073 Å
Hall symbol: -P 2ybc	Cell parameters from 19383 reflections
*a* = 7.2467 (14) Å	θ = 3.0–27.6º
*b* = 14.171 (3) Å	µ = 0.94 mm^−^^1^
*c* = 23.813 (5) Å	*T* = 293 (2) K
β = 97.11 (3)º	Block, black
*V* = 2426.6 (9) Å^3^	0.22 × 0.20 × 0.15 mm
*Z* = 2	

### Data collection

**Table:**

Bruker SMART CCD diffractometer	3886 reflections with *I* > 2σ(*I*)
Radiation source: fine-focus sealed tube	*R*_int_ = 0.073
Monochromator: graphite	θ_max_ = 27.5º
*T* = 293(2) K	θ_min_ = 3.0º
ω scans	*h* = −9→9
Absorption correction: none	*k* = −18→17
23688 measured reflections	*l* = −30→30
5568 independent reflections	

### Refinement

**Table:**

Refinement on *F*^2^	Secondary atom site location: difference Fourier map
Least-squares matrix: full	Hydrogen site location: inferred from neighbouring sites
*R*[*F*^2^ > 2σ(*F*^2^)] = 0.059	H-atom parameters constrained
*wR*(*F*^2^) = 0.115	*w* = 1/[σ^2^(*F*_o_^2^) + (0.0338*P*)^2^ + 2.4261*P*] where *P* = (*F*_o_^2^ + 2*F*_c_^2^)/3
*S* = 1.05	(Δ/σ)_max_ = 0.001
5568 reflections	Δρ_max_ = 0.41 e Å^−^^3^
316 parameters	Δρ_min_ = −0.35 e Å^−^^3^
Primary atom site location: structure-invariant direct methods	Extinction correction: none

### Special details

**Table:**

Geometry. All esds (except the esd in the dihedral angle between two l.s. planes) are estimated using the full covariance matrix. The cell esds are taken into account individually in the estimation of esds in distances, angles and torsion angles; correlations between esds in cell parameters are only used when they are defined by crystal symmetry. An approximate (isotropic) treatment of cell esds is used for estimating esds involving l.s. planes.
Refinement. Refinement of *F*^2^ against ALL reflections. The weighted *R*-factor *wR* and goodness of fit *S* are based on *F*^2^, conventional *R*-factors *R* are based on *F*, with *F* set to zero for negative *F*^2^. The threshold expression of *F*^2^ > σ(*F*^2^) is used only for calculating *R*-factors(gt) *etc*. and is not relevant to the choice of reflections for refinement. *R*-factors based on *F*^2^ are statistically about twice as large as those based on *F*, and *R*- factors based on ALL data will be even larger.

### Fractional atomic coordinates and isotropic or equivalent isotropic displacement parameters (Å^2^)

**Table:**

	*x*	*y*	*z*	*U*_iso_*/*U*_eq_	
Cu1	1.13786 (5)	0.05253 (3)	0.990416 (16)	0.02737 (12)	
N1	0.9279 (4)	0.2080 (2)	0.83101 (12)	0.0481 (8)	
H1	0.9928	0.1944	0.8612	0.058*	
N2	1.0262 (4)	0.3315 (2)	1.06884 (14)	0.0492 (8)	
H2	1.0768	0.2917	1.0500	0.059*	
O1	0.7459 (3)	0.00204 (19)	0.93852 (11)	0.0549 (7)	
O2	0.9804 (3)	0.09287 (18)	0.92135 (9)	0.0424 (6)	
O3	1.0252 (3)	0.15225 (16)	1.03190 (10)	0.0436 (6)	
O4	0.7885 (3)	0.06096 (16)	1.04750 (12)	0.0532 (7)	
O5	1.3926 (3)	0.12326 (16)	0.97707 (10)	0.0460 (6)	
H5A	1.4998	0.0999	0.9789	0.055*	
C1	0.8163 (4)	0.0628 (2)	0.90930 (13)	0.0319 (7)	
C2	0.6955 (4)	0.0996 (2)	0.85929 (13)	0.0311 (7)	
C3	0.5154 (5)	0.0638 (2)	0.84879 (14)	0.0404 (8)	
H3	0.4795	0.0165	0.8723	0.049*	
C4	0.3890 (5)	0.0955 (3)	0.80525 (16)	0.0502 (10)	
H4	0.2712	0.0688	0.7983	0.060*	
C5	0.4416 (5)	0.1681 (3)	0.77200 (16)	0.0536 (11)	
H5	0.3564	0.1922	0.7432	0.064*	
C6	0.6177 (5)	0.2053 (3)	0.78080 (15)	0.0473 (10)	
H6	0.6491	0.2544	0.7578	0.057*	
C7	0.7506 (4)	0.1714 (2)	0.82325 (14)	0.0354 (8)	
C8	1.0119 (5)	0.2737 (2)	0.79817 (15)	0.0391 (8)	
C9	1.1365 (5)	0.3380 (3)	0.82540 (16)	0.0464 (9)	
H9	1.1578	0.3381	0.8647	0.056*	
C10	1.2290 (6)	0.4012 (3)	0.7956 (2)	0.0625 (12)	
H10	1.3126	0.4435	0.8147	0.075*	
C11	1.1986 (7)	0.4023 (4)	0.7379 (2)	0.0776 (15)	
H11	1.2604	0.4456	0.7175	0.093*	
C12	1.0764 (6)	0.3393 (4)	0.71023 (19)	0.0752 (15)	
H12	1.0554	0.3403	0.6709	0.090*	
C13	0.9836 (5)	0.2743 (3)	0.73948 (16)	0.0544 (11)	
H13	0.9027	0.2312	0.7200	0.065*	
C14	0.8729 (4)	0.1384 (2)	1.05159 (13)	0.0323 (7)	
C15	0.7840 (4)	0.2162 (2)	1.07961 (13)	0.0308 (7)	
C16	0.6150 (5)	0.1969 (3)	1.10022 (14)	0.0395 (8)	
H16	0.5680	0.1358	1.0971	0.047*	
C17	0.5161 (5)	0.2646 (3)	1.12476 (16)	0.0454 (9)	
H17	0.4044	0.2499	1.1382	0.055*	
C18	0.5858 (5)	0.3544 (3)	1.12903 (16)	0.0485 (10)	
H18	0.5195	0.4013	1.1451	0.058*	
C19	0.7510 (5)	0.3763 (2)	1.11009 (16)	0.0441 (9)	
H19	0.7934	0.4383	1.1130	0.053*	
C20	0.8583 (4)	0.3083 (2)	1.08640 (14)	0.0353 (8)	
C21	1.1236 (5)	0.4172 (2)	1.08019 (18)	0.0446 (9)	
C22	1.2051 (5)	0.4608 (3)	1.03751 (19)	0.0583 (11)	
H22	1.1940	0.4345	1.0015	0.070*	
C23	1.3035 (6)	0.5441 (3)	1.0488 (2)	0.0743 (14)	
H23	1.3570	0.5738	1.0200	0.089*	
C24	1.3229 (6)	0.5830 (3)	1.1018 (3)	0.0756 (15)	
H24	1.3891	0.6388	1.1089	0.091*	
C25	1.2444 (6)	0.5391 (3)	1.1442 (2)	0.0630 (12)	
H25	1.2570	0.5656	1.1802	0.076*	
C26	1.1472 (5)	0.4564 (3)	1.13402 (18)	0.0520 (10)	
H26	1.0968	0.4264	1.1634	0.062*	
C27	1.4132 (6)	0.2166 (3)	0.95906 (19)	0.0658 (12)	
H27A	1.5422	0.2292	0.9568	0.099*	
H27B	1.3682	0.2592	0.9856	0.099*	
H27C	1.3433	0.2252	0.9225	0.099*	

### Atomic displacement parameters (Å^2^)

**Table:**

	*U*^11^	*U*^22^	*U*^33^	*U*^12^	*U*^13^	*U*^23^
Cu1	0.0269 (2)	0.0242 (2)	0.0313 (2)	−0.00364 (18)	0.00475 (14)	0.00085 (17)
N1	0.0402 (18)	0.061 (2)	0.0409 (18)	−0.0093 (16)	−0.0023 (14)	0.0202 (15)
N2	0.0371 (17)	0.0344 (17)	0.080 (2)	−0.0060 (14)	0.0218 (16)	−0.0171 (16)
O1	0.0446 (15)	0.0644 (18)	0.0516 (16)	−0.0184 (14)	−0.0104 (12)	0.0285 (14)
O2	0.0358 (14)	0.0535 (15)	0.0366 (14)	−0.0067 (12)	−0.0015 (11)	0.0123 (12)
O3	0.0407 (14)	0.0362 (14)	0.0571 (16)	−0.0074 (12)	0.0189 (12)	−0.0157 (12)
O4	0.0511 (16)	0.0295 (14)	0.085 (2)	−0.0092 (13)	0.0326 (14)	−0.0149 (13)
O5	0.0321 (13)	0.0390 (14)	0.0679 (18)	−0.0078 (11)	0.0098 (12)	0.0084 (13)
C1	0.0351 (19)	0.0304 (18)	0.0307 (17)	0.0024 (16)	0.0060 (14)	−0.0032 (15)
C2	0.0326 (18)	0.0308 (18)	0.0304 (17)	0.0025 (15)	0.0059 (14)	−0.0027 (14)
C3	0.038 (2)	0.042 (2)	0.041 (2)	−0.0035 (17)	0.0059 (16)	0.0038 (17)
C4	0.032 (2)	0.068 (3)	0.049 (2)	−0.0077 (19)	−0.0012 (17)	0.006 (2)
C5	0.037 (2)	0.077 (3)	0.045 (2)	0.009 (2)	−0.0025 (17)	0.017 (2)
C6	0.041 (2)	0.056 (2)	0.045 (2)	0.0051 (19)	0.0051 (17)	0.0188 (18)
C7	0.0319 (19)	0.040 (2)	0.0345 (19)	0.0012 (16)	0.0052 (14)	0.0004 (15)
C8	0.0348 (19)	0.040 (2)	0.042 (2)	0.0010 (17)	0.0046 (16)	0.0095 (16)
C9	0.046 (2)	0.047 (2)	0.045 (2)	−0.0055 (19)	0.0005 (17)	−0.0036 (18)
C10	0.053 (3)	0.046 (3)	0.088 (4)	−0.013 (2)	0.006 (2)	0.003 (2)
C11	0.064 (3)	0.083 (4)	0.085 (4)	−0.021 (3)	0.006 (3)	0.044 (3)
C12	0.055 (3)	0.120 (4)	0.048 (3)	−0.018 (3)	0.000 (2)	0.035 (3)
C13	0.042 (2)	0.074 (3)	0.046 (2)	−0.017 (2)	0.0003 (18)	0.010 (2)
C14	0.0312 (18)	0.0302 (19)	0.0347 (19)	−0.0006 (15)	0.0004 (14)	−0.0009 (14)
C15	0.0272 (17)	0.0312 (18)	0.0335 (18)	0.0007 (14)	0.0014 (14)	−0.0026 (14)
C16	0.039 (2)	0.035 (2)	0.045 (2)	−0.0044 (16)	0.0083 (16)	−0.0021 (16)
C17	0.035 (2)	0.045 (2)	0.060 (2)	−0.0055 (18)	0.0168 (18)	−0.0112 (19)
C18	0.033 (2)	0.047 (2)	0.066 (3)	0.0111 (18)	0.0081 (18)	−0.014 (2)
C19	0.035 (2)	0.0287 (19)	0.068 (3)	0.0007 (16)	0.0032 (18)	−0.0070 (17)
C20	0.0268 (18)	0.0327 (19)	0.046 (2)	0.0006 (15)	0.0031 (15)	−0.0048 (16)
C21	0.0280 (19)	0.0310 (19)	0.075 (3)	−0.0015 (16)	0.0094 (18)	−0.0045 (18)
C22	0.047 (2)	0.052 (3)	0.074 (3)	−0.005 (2)	0.003 (2)	0.006 (2)
C23	0.058 (3)	0.056 (3)	0.107 (4)	−0.018 (2)	0.003 (3)	0.027 (3)
C24	0.057 (3)	0.037 (2)	0.128 (5)	−0.011 (2)	−0.007 (3)	−0.003 (3)
C25	0.043 (2)	0.048 (3)	0.096 (4)	0.000 (2)	0.001 (2)	−0.023 (2)
C26	0.037 (2)	0.044 (2)	0.077 (3)	−0.0041 (19)	0.0122 (19)	−0.012 (2)
C27	0.074 (3)	0.046 (2)	0.077 (3)	−0.016 (2)	0.007 (2)	0.014 (2)

### Geometric parameters (Å, °)

**Table:**

Cu1—O4^i^	1.951 (2)	C9—H9	0.9300
Cu1—O1^i^	1.954 (2)	C10—C11	1.365 (6)
Cu1—O3	1.959 (2)	C10—H10	0.9300
Cu1—O2	1.967 (2)	C11—C12	1.367 (6)
Cu1—O5	2.159 (2)	C11—H11	0.9300
Cu1—Cu1^i^	2.5774 (10)	C12—C13	1.379 (5)
N1—C7	1.377 (4)	C12—H12	0.9300
N1—C8	1.402 (4)	C13—H13	0.9300
N1—H1	0.8314	C14—C15	1.477 (4)
N2—C20	1.375 (4)	C15—C16	1.402 (4)
N2—C21	1.413 (4)	C15—C20	1.413 (4)
N2—H2	0.8323	C16—C17	1.371 (5)
O1—C1	1.254 (4)	C16—H16	0.9300
O1—Cu1^i^	1.954 (2)	C17—C18	1.368 (5)
O2—C1	1.263 (4)	C17—H17	0.9300
O3—C14	1.266 (4)	C18—C19	1.366 (5)
O4—C14	1.255 (4)	C18—H18	0.9300
O4—Cu1^i^	1.951 (2)	C19—C20	1.400 (5)
O5—C27	1.404 (4)	C19—H19	0.9300
O5—H5A	0.8409	C21—C22	1.383 (5)
C1—C2	1.483 (4)	C21—C26	1.388 (5)
C2—C3	1.394 (5)	C22—C23	1.387 (6)
C2—C7	1.420 (4)	C22—H22	0.9300
C3—C4	1.371 (5)	C23—C24	1.368 (7)
C3—H3	0.9300	C23—H23	0.9300
C4—C5	1.380 (5)	C24—C25	1.369 (6)
C4—H4	0.9300	C24—H24	0.9300
C5—C6	1.373 (5)	C25—C26	1.374 (5)
C5—H5	0.9300	C25—H25	0.9300
C6—C7	1.392 (5)	C26—H26	0.9300
C6—H6	0.9300	C27—H27A	0.9600
C8—C9	1.386 (5)	C27—H27B	0.9600
C8—C13	1.387 (5)	C27—H27C	0.9600
C9—C10	1.369 (5)		
			
O4^i^—Cu1—O1^i^	87.77 (12)	C11—C10—H10	120.0
O4^i^—Cu1—O3	169.37 (10)	C9—C10—H10	120.0
O1^i^—Cu1—O3	90.61 (12)	C10—C11—C12	119.4 (4)
O4^i^—Cu1—O2	90.93 (12)	C10—C11—H11	120.3
O1^i^—Cu1—O2	169.23 (10)	C12—C11—H11	120.3
O3—Cu1—O2	88.69 (11)	C11—C12—C13	121.3 (4)
O4^i^—Cu1—O5	91.53 (10)	C11—C12—H12	119.3
O1^i^—Cu1—O5	91.52 (10)	C13—C12—H12	119.3
O3—Cu1—O5	99.02 (10)	C12—C13—C8	119.6 (4)
O2—Cu1—O5	99.21 (10)	C12—C13—H13	120.2
O4^i^—Cu1—Cu1^i^	82.43 (8)	C8—C13—H13	120.2
O1^i^—Cu1—Cu1^i^	83.02 (8)	O4—C14—O3	123.1 (3)
O3—Cu1—Cu1^i^	86.94 (7)	O4—C14—C15	116.9 (3)
O2—Cu1—Cu1^i^	86.21 (7)	O3—C14—C15	120.0 (3)
O5—Cu1—Cu1^i^	171.98 (7)	C16—C15—C20	118.5 (3)
C7—N1—C8	129.6 (3)	C16—C15—C14	117.5 (3)
C7—N1—H1	116.6	C20—C15—C14	124.0 (3)
C8—N1—H1	113.4	C17—C16—C15	122.6 (3)
C20—N2—C21	125.9 (3)	C17—C16—H16	118.7
C20—N2—H2	117.8	C15—C16—H16	118.7
C21—N2—H2	116.2	C18—C17—C16	118.4 (3)
C1—O1—Cu1^i^	126.2 (2)	C18—C17—H17	120.8
C1—O2—Cu1	121.6 (2)	C16—C17—H17	120.8
C14—O3—Cu1	120.8 (2)	C19—C18—C17	121.2 (3)
C14—O4—Cu1^i^	126.8 (2)	C19—C18—H18	119.4
C27—O5—Cu1	127.4 (2)	C17—C18—H18	119.4
C27—O5—H5A	104.8	C18—C19—C20	122.0 (3)
Cu1—O5—H5A	127.6	C18—C19—H19	119.0
O1—C1—O2	122.8 (3)	C20—C19—H19	119.0
O1—C1—C2	116.5 (3)	N2—C20—C19	121.0 (3)
O2—C1—C2	120.6 (3)	N2—C20—C15	121.7 (3)
C3—C2—C7	118.6 (3)	C19—C20—C15	117.3 (3)
C3—C2—C1	117.6 (3)	C22—C21—C26	119.0 (4)
C7—C2—C1	123.7 (3)	C22—C21—N2	119.5 (4)
C4—C3—C2	122.6 (3)	C26—C21—N2	121.5 (4)
C4—C3—H3	118.7	C21—C22—C23	119.6 (4)
C2—C3—H3	118.7	C21—C22—H22	120.2
C3—C4—C5	118.2 (3)	C23—C22—H22	120.2
C3—C4—H4	120.9	C24—C23—C22	120.9 (5)
C5—C4—H4	120.9	C24—C23—H23	119.6
C6—C5—C4	121.1 (3)	C22—C23—H23	119.6
C6—C5—H5	119.5	C23—C24—C25	119.6 (4)
C4—C5—H5	119.5	C23—C24—H24	120.2
C5—C6—C7	121.6 (4)	C25—C24—H24	120.2
C5—C6—H6	119.2	C24—C25—C26	120.5 (5)
C7—C6—H6	119.2	C24—C25—H25	119.7
N1—C7—C6	121.1 (3)	C26—C25—H25	119.7
N1—C7—C2	121.1 (3)	C25—C26—C21	120.4 (4)
C6—C7—C2	117.7 (3)	C25—C26—H26	119.8
C9—C8—C13	118.2 (3)	C21—C26—H26	119.8
C9—C8—N1	118.5 (3)	O5—C27—H27A	109.5
C13—C8—N1	123.1 (3)	O5—C27—H27B	109.5
C10—C9—C8	121.4 (4)	H27A—C27—H27B	109.5
C10—C9—H9	119.3	O5—C27—H27C	109.5
C8—C9—H9	119.3	H27A—C27—H27C	109.5
C11—C10—C9	120.1 (4)	H27B—C27—H27C	109.5

Symmetry codes: (i) −*x*+2, −*y*, −*z*+2.

### Hydrogen-bond geometry (Å, °)

**Table:**

*D*—H···*A*	*D*—H	H···*A*	*D*···*A*	*D*—H···*A*
N1—H1···O2	0.83	2.04	2.690 (4)	135
N2—H2···O3	0.83	2.05	2.688 (4)	133
O5—H5A···O1^ii^	0.84	2.54	3.306 (4)	152
O5—H5A···O4^ii^	0.84	2.55	3.260 (4)	143

Symmetry codes: (ii) *x*+1, *y*, *z*.

## References

[bb1] Bruker (1998). *SAINT-Plus* and *SMART* Bruker AXS Inc., Madison, Wisconsin, USA.

[bb2] Burnett, M. N. & Johnson, C. K. (1996). *ORTEPIII* Report ORNL-6895. Oak Ridge National Laboratory, Tennessee, USA.

[bb3] Churchill, M. R., Li, Y.-J., Nalewajek, D., Schaber, P. M. & Dorfamanii, J. (1985). *Inorg. Chem.* **24**, 2684–2687.

[bb4] Facchin, G., Torre, M. H., Kremer, E., Piro, O. E. & Baran, E. J. (1998). *Z. Anorg. Allg. Chem.* **53**, 871–874.

[bb5] Farrugia, L. J. (1997). *J. Appl. Cryst.* **30**, 565.

[bb6] Martin, J. D. & Greenwood, K. B. (1997). *Angew. Chem. Int. Ed.* **36**, 2072–2075.

[bb7] Melnik, M., Koman, M. & Glowiak, T. (1998). *Polyhedron*, **17**, 1767–1771.

[bb8] Moulton, B., Abourahma, H., Bradner, M. W., Lu, J.-J., McManus, G. J. & Zaworotko, M. J. (2003). *Chem. Commun.* pp. 1342–1343. 10.1039/b301221b 12841232

[bb9] Sheldrick, G. M. (2008). *Acta Cryst.* A**64**, 112–122. 10.1107/S0108767307043930 18156677

